# COVID-19: Could India still escape?

**DOI:** 10.7189/jogh.10.010372

**Published:** 2020-06

**Authors:** Ashwin Rammohan, Mohamed Rela

**Affiliations:** The Institute of Liver Disease and Transplantation, Dr. Rela Institute and Medical Centre, Bharat Institute of Higher Education and Research, Chennai, India

There appear to be numerous reasons, both natural and man-made, which could soften the “COVID-19 blow” on India. This is something that has not been addressed previously, and our manuscript critically analyzes the reasons why India could still come out of the COVID-19 pandemic relatively unscathed, thereby providing new insights into an aspect of the pandemic and lessons for the future.

It is a bit of a mystery how the world’s second-most-populous nation, with 1.3 billion people, has remained relatively unaffected while the number of deaths explode to its east and west. This has spawned a sense of disbelief about the crisis in some quarters. Due to its selective testing policy, there is likely to be a reporting bias, with many more cases in India that have not been detected [1-3]. Although it is indeed notable that notwithstanding an initial sluggish approach, India has been testing over 100 000 samples a day [4]. Even with testing being ramped up to numbers close to those of the western countries, given that India has 20% of the world’s population, it may not be enough. To put into perspective, if tests were performed at the USA’s current testing numbers, it would take 15 years to assay the Indian population once! Nonetheless, if indeed this anomaly in numbers was due to a deficit in reporting, then there should have been a spike in the ICU admissions and mortality rates, which remarkably, has remained relatively low over the past three months. Using the COVID-19 mortality rate as a surrogate yardstick for numbers, rates in India are among the lowest in the world (India 3.1% vs China 5.6%, USA 6% and global average 6.5%) [2,5,6]. It is also remarkable to note that as compared to the same time in 2019, the actual number of deaths in large cities like Mumbai and Ahmedabad fell by 21% and 67% respectively; most likely a serendipitous effect of the COVID-19 lockdown measures [2,6].

Certain other countries like Singapore, Germany and South Korea too have shown low mortality rates, however their deaths/million population is much higher (0.04 vs 0.8-11) [5]. Taking the example of Germany, the reasons are clearer; the affected population were those who returned from Italian ski-trips and were naturally healthier and younger. Germany also has successfully been able to insulate their vulnerable population, and have the added luxury of sufficient ICU beds and respirators [7]. The scenario in India could not be more distinct from that of Germany. Despite this, the dreaded “3^rd^ week surge” of COVID-19 cases, which consists of an explosive increase numbers, as observed in Italy, Iran and Spain has been conspicuous by its absence in India. Consequent to the difficulties of getting tested, or possibly because of quick and strict efforts right from the start, has India indeed managed to so far escape the worst? Complacency at this stage, however, may lead to a “second-wind” for the virus leading to a late peak in cases, and needs to be strictly guarded against.

There may be certain reasons why the virus has not caused the same degree of damage so far. India has a decent track record with epidemics, it has contained flu epidemics in the past. From eradication of small-pox in the 1980s to successfully vaccinating 170 million to effectively eradicate polio in 2016, mass community health drives are not new to India [8]. Malaria is endemic and antimalarials like Chloroquine are often started empirically for resistant fever. The same is the case with Macrolide antibiotics like Azithromycin for lower respiratory tract infections. Unsurprisingly, these two drugs, which purportedly have anti-coronavirus action and are under trial, could have played a role in serendipitously controlling the disease. India is one of the world’s largest producers of generic drugs. Antibiotics are often dispensed without prescriptions, and are available over-the-counter. Many of the people dying from the coronavirus succumb to secondary infections, and some of those can be treated/prevented with antibiotics. Demographics also work to India’s advantage. The population here is considerably younger than in the countries worst hit, giving them a better chance of coping with the infection. A lesser factor is that India’s climate is warmer, with temperatures hovering around the mid 30°C to mid 40°C, it is likely to have deterred the virus. This though, has not been clinically proven to have a major effect. There are unpublished studies that suggest that the virus may have mutated into a less virulent strain, and that universal BCG vaccination has provided an immunological advantage, leading to a lower incidence of infections in the Indian subcontinent. These papers would however need peer-review and validation, before being accepted [2,9,10].

This optimism however needs to be tempered. The sobering thought that if, in fact the above hypotheses are wrong, then India with its archaic public health system, one of the lowest per capita ICU bed ratios in the world (2.3beds/100 000 population), lack of adequately trained personnel and a large impoverished rural and slum population, will hopelessly careen towards a catastrophic health crisis [3,11,12]. It will be an economic and social disaster, from which India might take decades to come out of.

India has also been proactive in announcing preventive measures. It was one of the first nations to essentially close its international and regional borders, cancelling visas and denying entry to all but a select few foreigners. Thermal screening was set up at air and sea ports, with universal WHO based screening for all disembarking passengers being mandatory. Indians stranded across the world from Wuhan to Maldives have been evacuated, quarantined, tested and discharged when negative. Southern states, such as Kerala and Tamilnadu have essentially shut down domestic borders, temperature tests are done to screen those in cars and trains. Kerala has drawn on its experience with the Nipah virus in 2018 to use extensive testing, contact tracing, and community mobilisation to contain the contagion. It has also set up thousands of temporary shelters for migrant workers. Odisha’s experience with natural disasters meant crisis precautions were already in place and these have now been repurposed. Massive hygiene and “social-isolation” campaigns have been launched on the electronic media, radios and phones, educating the community on the magnitude of the problem at hand and their role in fighting it [2,3,6].

The largest COVID-19 national lockdown in the world came into force on Mar 24, 2020, which WHO praised as “tough and timely”. What was initially a three-week lockdown, has sequentially been extended with varying degrees of measures till at least May 31, 2020. The central government has proactively announced and effectively enforced the lockdown, baring the civic, health and other essential services; completely shutting down India Inc. Religious places, schools and universities have closed, so have swimming pools, gyms, malls and movie theatres. Public gathering including weddings are banned. Certain states are enforcing laws to make these illegal, with the police ensuring truant members of the public are dealt with appropriately. Another notable initiative has been the effort to combat “fake news” which is likely to fuel mass hysteria. A group of more than 400 multidisciplinary Indian scientists, have voluntarily formed Indian Scientists’ Response to COVID-19 to fight myths and misinformation about the disease [2,3,6,11].

The next step has been to prepare for a delayed and more protracted version of the “3rd week surge”. There has been an increase in the number of the research laboratories approved for testing the virus. Government directives and guidelines have been released to provide for measures and infrastructural changes to hospitals to tackle the likely explosion in cases over the fortnight. Private and public sector hospitals have been gearing up with stringent screening measures, ER-triage areas, and dedicated isolated COVID-19 floors with beds and ICU. Certain states like Tamilnadu and Karnataka have designated public-sector hospitals to ensure centralisation of resources, thereby tackling the surge more effectively in a cohesive manner. These hospitals are being stocked and staffed appropriately. Healthcare workers are being trained to work efficiently and safely during the crisis. The Department of Pharmaceuticals submitted a report that the existing stock will be sufficient to manufacture drugs for two to three months. With the epidemiccurve apparently flattened, the aim now remains to keep it down. The immediate challenge is to keep infections at manageable levels and ensure the ability to test, trace contacts, isolate patients, implement COVID care plans, and disseminate timely information.

**Figure:**
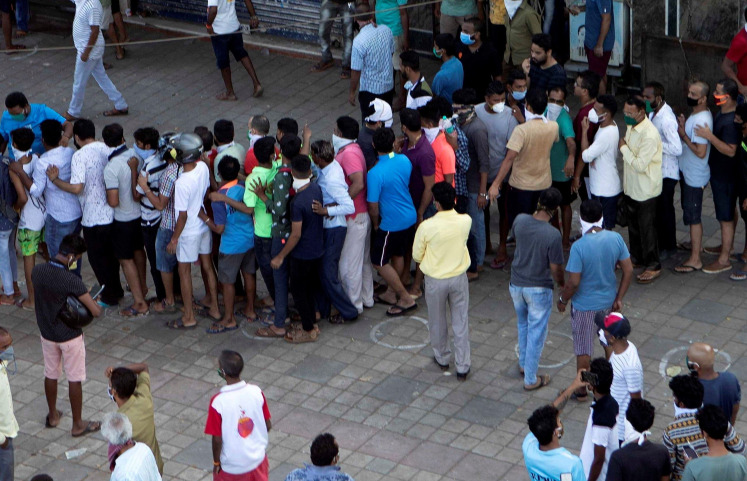
Photo: People violate social distancing norms (white circles) as they stand in queue to buy alcohol after authorities permitted the opening of liquor stores during the ongoing COVID-19 nationwide lockdown (used with permission).

We are in an unprecedented global war, and are facing a single common enigmatic enemy, the novel coronavirus. To ensure this war is won, it is imperative that there is awareness of the clear and present danger to humanity at large and that the medical staff is guaranteed sufficient resources, including training and technology [1]. This fight against the virus is unlikely to be short-lived, any victories now must be viewed with guarded pragmatism. Changing seasons, lack of unlimited resources, mutating viruses, and an economic depression looming in the background are likely to make this war a protracted one. At this moment however, sharing our experiences and lessons, is the only chance to win. In the absence of validated data from trials, there is strong dependence on experience based on previous similar epidemics, and from consensus based on expert opinions. This pandemic does come with the silver lining of worldwide collaboration in clinical care and biomedical research. These inter-country associations with high quality research and accurate documentation may help speed up discoveries and our capacity to fight the virus. Nevertheless, India’s population of 1.3 billion across diverse states, health inequalities, wide economic and social disparities, and distinct cultural values present unique challenges. Identification of gaps in our current knowledge and actions will help resolve the most burning issues, to drive the pathways of discovery and recovery. India appears to have taken bold pre-emptive steps in this battle, only time will confirm if they are in the right direction.
